# Heart Murmur Classification Using a Capsule Neural Network

**DOI:** 10.3390/bioengineering10111237

**Published:** 2023-10-24

**Authors:** Yu-Ting Tsai, Yu-Hsuan Liu, Zi-Wei Zheng, Chih-Cheng Chen, Ming-Chih Lin

**Affiliations:** 1 Master’s Program in Electro-Acoustics, Feng Chia University, Taichung 40724, Taiwan; 2 Hyper-Automation Laboratory, Feng Chia University, Taichung 40724, Taiwan; 3 Program of Mechanical and Aeronautical Engineering, Feng Chia University, Taichung 40724, Taiwan; 4 Department of Automatic Control Engineering, Feng Chia University, Taichung 40724, Taiwan; 5 Department of Post-Baccalaureate Medicine, College of Medicine, National Chung Hsing University, Taichung 402, Taiwan; 6 Children’s Medical Center, Taichung Veterans General Hospital, Taichung 40705, Taiwan; 7 School of Medicine, National Yang Ming Chiao Tung University, Taipei 112304, Taiwan; 8 Department of Food and Nutrition, Providence University, Taichung 433, Taiwan; 9 School of Medicine, Chung Shan Medical University, Taichung 40201, Taiwan

**Keywords:** heart murmur, auscultation, heart sound diagnosis, cardiac dysphonic diagnosis, capsule neural network, deep learning in healthcare

## Abstract

The healthcare industry has made significant progress in the diagnosis of heart conditions due to the use of intelligent detection systems such as electrocardiograms, cardiac ultrasounds, and abnormal sound diagnostics that use artificial intelligence (AI) technology, such as convolutional neural networks (CNNs). Over the past few decades, methods for automated segmentation and classification of heart sounds have been widely studied. In many cases, both experimental and clinical data require electrocardiography (ECG)-labeled phonocardiograms (PCGs) or several feature extraction techniques from the mel-scale frequency cepstral coefficient (MFCC) spectrum of heart sounds to achieve better identification results with AI methods. Without good feature extraction techniques, the CNN may face challenges in classifying the MFCC spectrum of heart sounds. To overcome these limitations, we propose a capsule neural network (CapsNet), which can utilize iterative dynamic routing methods to obtain good combinations for layers in the translational equivariance of MFCC spectrum features, thereby improving the prediction accuracy of heart murmur classification. The 2016 PhysioNet heart sound database was used for training and validating the prediction performance of CapsNet and other CNNs. Then, we collected our own dataset of clinical auscultation scenarios for fine-tuning hyperparameters and testing results. CapsNet demonstrated its feasibility by achieving validation accuracies of 90.29% and 91.67% on the test dataset.

## 1. Introduction

Cardiovascular diseases (CVDs) remain a major global health concern that causes significant numbers of deaths and morbidities. In clinical practice, auscultation is one of the non-invasive methods for assessing the cardiovascular system and detecting pathological cardiac conditions, such as arrhythmias, valve disorders, and heart failure. Heart sounds serve as a valuable initial indicator for evaluating diseases, guiding further diagnostic tests, and playing a pivotal role in the early detection of CVDs. The PCG recordings [1] of these sounds, obtained through sensors on the chest surface, provide a useful tool for clinicians to gain insights into the heart’s health, enabling timely intervention and reducing the risks of mortality and morbidity due to CVDs. In recent years, there have been numerous studies on the use of machine learning techniques for heart disease detection and diagnosis based on PCG recordings, such as cardiac dysphonic diagnosis [1,2] and medical-grade artificial intelligence technology [3,4], and their development statuses have been discussed [5]. Kumar et al. [6] presented a method for classifying heart murmurs that involved feature extraction, feature selection, and classification using a nonlinear classifier. The authors suggested a new set of 17 features extracted in the time, frequency, and state space domains. These features were then reduced to 10 features using sequential forward feature selection (SFFS). Vepa [7] investigated the use of features derived from the cepstrum of heart sound signals to classify murmurs into normal, systolic, and diastolic using a support vector machine (SVM) [7,8] trained on cepstral features. Huang et al. [9] aimed to develop an intelligent diagnostic method for detecting heart murmurs in patients with ventricular and atrial septal defects. Shekhar et al. [10] developed a computer algorithm to assist primary care providers in identifying Still’s murmur in children, thereby decreasing overreferral to pediatric cardiologists. Additionally, there have been studies on the use of machine learning techniques for heart disease prediction, such as the ANN-based approach [2] for the detection and identification of congenital heart disease in pediatric patients and the cardiovascular disease prediction model based on the improved deep belief network [11]. To improve the generalization and robustness against noise, the use of a deep learning network model [12] and a complete ensemble empirical mode decomposition method [13] have also been proposed. A large annotated dataset and a very deep convolutional network [14,15], which can map a sequence of ECG samples to a sequence of arrhythmia annotations, are key to the performance of these models. Although there are still challenges to overcome in applying machine learning techniques to heart disease diagnosis [1,16,17,18] and prediction, these studies show promise in improving the accuracy and efficiency of cardiovascular care [18,19]. The 2016 PhysioNet/Computing in Cardiology Challenge [20,21] is a large collection database with several cardiac audio signal datasets used for heart murmur training, which aims to facilitate research on novel methods for heart sound classification. The mean accuracy of the convolutional neural network on this dataset was 86.8%.

The capsule neural network (CapsNet) has been widely used in various medical fields in recent years. CapsNet [22,23] is a type of machine learning system that is a variation of an artificial neural network. Its aim is to improve the modeling of hierarchical relationships by more closely imitating biological neural organization. CapsNet exhibits advantages over convolutional neural networks (CNNs) in modeling part-to-whole relationships between entities and learning viewpoint-invariant representations. This approach involves adding “capsules” to a convolutional neural network and using output from multiple capsules to create more stable representations for higher capsules. The output of CapsNet is a vector composed of the probability of an observation and its pose, which resembles the process used for classification with localization in CNNs. A variety of improved designs and applications [24] of capsule networks have been explored within the past decade, especially applications in medical images [25]. Lei et al. [26] proposed a model based on CapsNet to process medical images and achieved sufficient diagnostic results, highlighting the potential of CapsNet in noise reduction and effective diagnosis. Butub et al. [27] successfully used CapsNet to automatically learn relevant features from electrocardiogram signals to achieve the automatic detection of coronary artery disease (CAD), further emphasizing the importance of CapsNet in medical applications. El Boujnouni et al. [28] combined a capsule network with the wavelet decomposition image method to automatically diagnose cardiovascular diseases, once again demonstrating the superiority of CapsNet in processing small datasets. In addition, a study on bladder cancer detection [29] clearly demonstrated that CapsNet could be trained from smaller datasets, which is very beneficial for medical imaging diagnosis.

This paper uses deep learning to establish an intelligent auscultation assistance system to aid in the diagnosis of heart murmurs. Auscultation is used to record the subjects’ heart sounds to represent the clinical situation more accurately. Among the many studies and results on this topic, we hope that our study will provide novel insights regarding the application of a capsule neural network (CapsNet) to diagnose heart murmurs. CapsNet uses vectors to analyze and detect features, identifying differences in the positions of components and assessing them with good results. To better address the translation equivariance of input features, we use the softmax function to emphasize spatial attention, highlighting energy disparities between time and frequencies in spectral images. The primary capsules are intensified to encode the presence of a particular feature as well as its location in the input MFCC spectrum. We compare the results of CapsNet with those of traditional convolutional neural networks in terms of recognizing abnormal heart sound features. The 2016 PhysioNet heart sound database is used to train and validate the prediction performance of CapsNet. Various convolutional neural networks are compared to CapsNet in this study. In addition, we collect our own dataset to fine-tune the hyperparameters and test results in clinical auscultation scenarios. CapsNet exhibits the best prediction ability, which fits our purposes of improving the precision and efficiency of heart disease diagnosis, providing doctors with more powerful aids, and providing patients with better diagnosis and treatment experiences.

## 2. Methodological Description of CapsNet

CapsNet is an extension of traditional convolutional neural networks designed to address some of their limitations. Compared to CNNs, CapsNet stores features as vectors and maintains the distances and corresponding relationships between them. This leads to more accurate classification, improved translation invariance, and the ability to learn with less data.

### 2.1. Data Preprocessing

**Figure 1:**
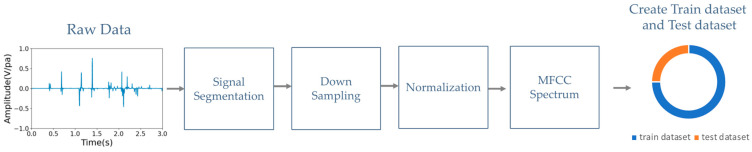
Flowchart of data preprocessing for the training and testing datasets.

Figure 1 shows the flowchart of preprocessing each heart sound to the MFCC spectrum for the training and testing datasets. This process involves four steps: signal segmentation, downsampling, normalization, and MFCC spectrum analysis.

(a) Signal segmentation: Each segment typically corresponds to a fixed time duration in seconds. The default time duration of each segment is 5 s, which corresponds to 220.5 k samples at a sampling rate of 44.1 kHz.
(b) Downsampling: Downsampling can be performed to decrease the computational load and storage requirements while preserving the essential information in the heart signal. Our downsampling rate was reduced from 44.1 kHz to 2 kHz, as in the 2016 PhysioNet heart sound database. With this downsampling rate, the integrity of heart sounds below 1 kHz is maintained.
(c) Normalization: This step is the process of scaling the heart sound signal to a standard range between −1 and 1 to prevent clipping or distortion.
(d) MFCC spectrum analysis: To extract features from the audio data, the mel-frequency cepstral coefficients (MFCCs) can be computed using a signal processing library such as librosa [30]. MFCCs capture important characteristics of audio signals, such as the spectral envelope and the spectral distribution of energy over time. The resulting MFCC spectrum can then be passed through the primary capsule layer of a CapsNet model to extract local features and encode them into capsule vectors. These capsule vectors can then be used to classify heart murmurs or other cardiac abnormalities. The CapsNet model offers the advantage of detecting multiple abnormalities simultaneously due to its ability to represent multiple features in a single capsule vector.

### 2.2. Methodology of CapsNet

**Figure 2:**
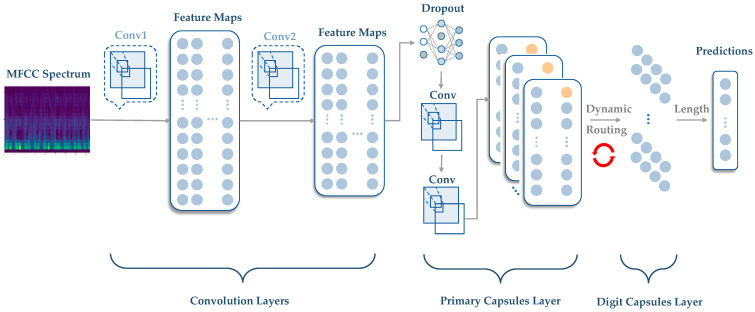
Model architecture of the capsule network.

Figure 2 shows the architecture of a standard capsule neural network, which consists of the main convolutional layers, primary and digit capsules (caps), and one fully connected layer. The primary caps layer is the first layer of CapsNet and is responsible for detecting local patterns in the input image. This layer consists of convolutional filters, each detecting a specific feature in the input image. The output of this layer is a set of “capsules”, each representing a particular feature and its associated probability. The digit caps layer is the second layer of CapsNet and is responsible for combining the information from the primary caps layer to produce the network’s final output. Each capsule in the primary caps layer is connected to every capsule in the digit caps layer, and the probability of the corresponding feature weights the connections between them. The output of the digit caps layer is a set of vectors, each representing a specific class’s parameters.

The primary capsule layer in CapsNet is responsible for extracting local features from the input data of the convolution layers and encoding them into capsule vectors. Dynamic routing is used in the primary capsule layer to determine the weights for combining the input data to form the capsule vectors. The dynamic routing algorithm for the primary capsule layer consists of the following steps:

(a) Routing initialization: The output of the primary capsule layer is a set of capsule vectors $\nu_{i,j,k}\in R^{D}$, where $H^{\prime }\times W^{\prime }\times N$ is the number of capsules in the primary capsule layer.

The convolutional layer applies a set of learnable filters to the input data to extract local features. The output of the convolutional layer is a set of feature maps, where each map corresponds to a different filter. The input data are denoted by $X\in R^{H\times W\times C}$, where H is the height, *W* is the width, *c* is the number of channels, and the convolutional layer has F filters of size K×K, with a stride of *S*. Then, the primary capsules $v_{i,j,k}$ encode the presence of a particular feature as well as its pose or location in the input data. The primary capsule layer often starts with a convolutional layer that extracts low-level features from the input data. The convolutional layer is represented by:

(1) $$\nu_{i,j,k}^{\left(0\right)}=\sigma \left(\sum_{u=0}^{K-1}\sum_{v=0}^{K-1}\sum_{c=0}^{c-1}w_{u,v,c,k}X_{S_{i+u},S_{j+v,c}}+b_{k}\right)$$

which is a set of $H^{\prime }\times W^{\prime }\times N$ capsule vectors $v_{i,j,k}\in R^{D}$, where $H^{\prime }=\left\lfloor \frac{H-K}{S}\right\rfloor +1$, $W^{\prime }=\left\lfloor \frac{W-K}{S}\right\rfloor +1$, s is the stride, *N* is the number of capsules per spatial location $\left(i,j\right)$, σ is the activation function (e.g., ReLU [31]), **w** is the weight of the *k*-th filter at position $\left(u,v\right)$ and channel c, and $b_{k}$ is the bias term for the *k*-th filter. These coefficients of capsule vectors are initialized randomly.

(b) Routing iteration: The routing algorithm iteratively updates the coupling coefficients based on the agreement between the capsule vectors and the output vectors of the higher layer capsules. The goal is to increase the coupling coefficients between capsules that are in agreement and decrease the coupling coefficients between capsules that are not in agreement.

The output vectors of the capsules in the layer above are normalized with a nonlinear activation function named Squash to ensure that they have a magnitude between 0 and 1. The squash function ensures that the length of the output vectors represents the probability that a specific feature or entity exists, and it is used in the routing process to determine how much information is passed between capsule vectors $\nu_{i,j,k}$. The squash equation is as follows:

(2) $$s_{i}^{\left(r\right)}=squash\left(\nu_{i}^{\left(r\right)}\right)=\frac{{\left\|\nu_{i}^{\left(r\right)}\right\|}^{2}}{1+{\left\|\nu_{i}^{\left(r\right)}\right\|}^{2}}\frac{\nu_{i}^{\left(r\right)}}{\left\|\nu_{i}^{\left(r\right)}\right\|}$$

where $v_{i}$ is the *i*-th capsule vector and $s_{i}^{\left(r\right)}$ is the squashed vector. The squashed capsule vectors are used to compute the prediction vectors for the higher-level capsules $u_{j|i}^{\left(r\right)}$ of the *j*-th higher layer capsule:

(3) $$u_{j|i}^{\left(r\right)}=W_{i,j}^{\left(r\right)}s_{i}^{\left(r\right)}$$

where $W_{i,j}^{\left(r\right)}$ is a weight matrix that maps the *i*-th capsule to the *j*-th capsule in the next layer.

To address the translation equivariance of input features, we use the softmax function to emphasize spatial attention, highlighting energy disparities between time and frequencies in spectral images. During the *r*-th iteration of the routing algorithm, the routing coefficients between the *i*-th primary capsule and the *j*-th higher layer capsule are calculated as follows:

(4) $$c_{ij}^{\left(r\right)}\;=\;softmax\left(b_{i,j}\right)=\;\frac{\exp\left(b_{i,j}^{\left(r\right)}\right)}{\sum_{n=1}^{N}\exp\left(b_{i,n}^{\left(r\right)}\right)\;}$$

where $b_{i,j}^{\left(r\right)}$ is the logit output of the agreement function between the *i*-th primary capsule and the *j*-th higher layer capsule, which is defined as:

(5) $$b_{i,j}^{\left(r\right)}=b_{i,j}^{\left(r-1\right)}+u_{j|i}^{\left(r\right)}\cdot \nu_{j}^{\left(r\right)}$$

Note that $b_{i,j}^{\left(0\right)}=0$. The weighted sum of the prediction vectors is then computed using the routing coefficients as follows:

(6) $$s_{j}^{\left(r\right)}=\sum_{i=1}^{M}c_{i,j}^{\left(r\right)}u_{j|i}^{\left(r\right)}$$

where *M* is the number of capsules in the digit capsule layer, $c_{i,j}^{\left(r\right)}$ is the coupling coefficient from Equation (4) at routing iteration *r*, and $s_{j}^{\left(r\right)}$ denotes the weighted sum of the logit values for all capsules in the digit capsule layer. Then, the output vector $\upsilon_{j}^{\left(r\right)}$ of capsule *j* is obtained by squashing again with the weighted sum of the prediction vectors:

(7) $$\upsilon_{j}^{\left(r\right)}=squash\left(s_{j}^{\left(r\right)}\right)=\frac{{\left\|s_{i}^{\left(r\right)}\right\|}^{2}}{1+{\left\|s_{i}^{\left(r\right)}\right\|}^{2}}\frac{s_{i}^{\left(r\right)}}{\left\|s_{i}^{\left(r\right)}\right\|}$$

The primary capsules output a vector of activations that indicate the presence of a feature at a particular location. These activations are fed into a nonlinear activation function, which digitizes them to produce a binary output.

As described in the above steps, the output of the primary capsule layer is input to the digital capsule layer, which is responsible for classification. The input of the digital capsule layer consists of the high-level feature vectors, and vectors for each class are the output. The length of these output vectors represents the probability of the input belonging to each class. The length of the output vectors is calculated using the Euclidean norm. The binary output represents the presence or absence of a feature at a particular location. ${\hat{v}}_{j}$ is the predicted output, with digitized values of 0 or 1 determined from the following equation:

(8) $$\hat{v_{c}}=\left\{\begin{array}{c} \;\;\;\;\;\;\;1,\;\;\;\;\;\mathrm{if}\;{∥v_{j}∥}^{2}>T^{2}\;\;\;\;\\ \;\;\;\;\;\;0,\;\;\;otherwise.\;\;\;\;\;\;\;\;\; \end{array}\right.$$

where $\left\|.\right\|$ is the Euclidean norm, and *T* is a digitization threshold.

The margin loss is then used to calculate the difference between the predicted output and the true label. This loss encourages a margin of at least a certain distance between the predicted output and the true label, which helps to prevent overfitting. The margin loss function is defined as follows:

(9) $$L_{2}=\sum_{c}T_{c}{\max\left(0,m^{+}-\left|v_{c}\right|\right)}^{2}+\lambda \left(1-T_{c}\right){\max\left(0,\left|v_{c}\right|-m^{-}\right)}^{2}$$

where $T_{c}$ is the true label of the c-th class, $m^{+}$ and $m^{-}$ are the upper and lower margins, respectively, and λ is a weighting parameter that balances the contributions of the two terms in the loss function. The digitized output $\hat{v_{c}}$ is calculated using Equation (8), where the Euclidean norm is denoted by $\left|\cdot \right|$. The margin loss function encourages a margin of at least $m^{+}$ between the predicted output and the true label when $T_{c}=1$ and a margin of at least $m^{-}$ between the predicted output and the true label when $T_{c}=0$. The loss function penalizes the deviation from these margins, squared and weighted by the parameters $T_{c}$ and λ. By minimizing the overall loss, the CapsNet model learns to accurately classify the input heart murmur spectrum into multiple classes using high-level feature vectors.

A summary of the CapsNet neural network architecture is listed in Table 1. Hyperparameters such as the learning rate, batch size, number of epochs, and optimizer play a crucial role in training and optimizing the performance of the proposed CapsNet architecture. The learning rate determines the step size at which the model’s weights are updated during training. The batch size determines the number of data samples processed in each iteration (minibatch) during training. The number of epochs specifies how many times the entire training dataset is passed forward and backward through the network. The optimizer is an algorithm that is responsible for updating the model’s weights during training to minimize the loss function. Adam is used as the optimizer in this study. The hyperparameter settings for training the CapsNet model are shown in Table 2, and we also discuss the different hyperparameter settings for optimizing the CapsNet performance in Section 3.3.

## 3. Experiments and Results

### 3.1. 2016 PhysioNet Heart Sound Database

**Figure 3:**
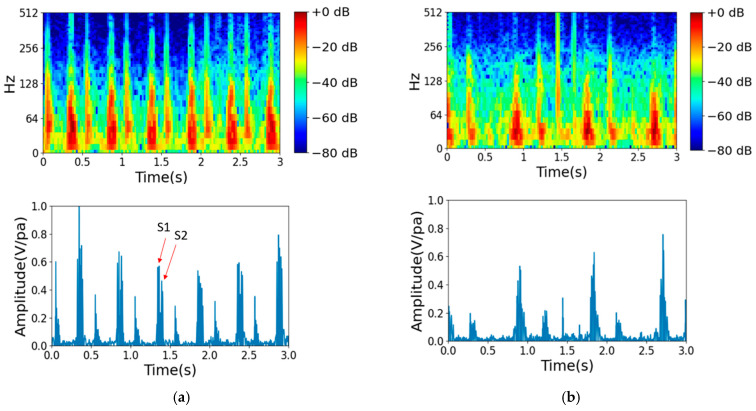
Examples from audio spectrum analysis of heart sounds from the 2016 PhysioNet heart sound dataset. (**a**) Normal case. (**b**) Abnormal case.

The 2016 PhysioNet Heart Sound database is a public database derived from heart sound recordings collected in various clinical or nonclinical (e.g., home visit) settings and contains 3251 recordings of both normal and abnormal heart sounds. Normal recordings come from healthy subjects, while abnormal recordings come from patients with confirmed heart disease. The heart sound recordings range from 5 s to over 120 s. It is crucial to understand the characteristics of heartbeats, especially the first and second heart sounds, represented as S1 and S2, respectively. These sounds can be heard as vibrations throughout the entire cardiac structure, which can be recorded as time series representations. The 2016 PhysioNet heart sound dataset provides valuable information on the typical frequency range for each type of heart sound, with murmurs having a diverse frequency range and respiration having a frequency range of 200–700 Hz. In this study, mel-frequency cepstral coefficient (MFCC) spectrum analysis was used to analyze the heart sound recordings. The MFCCs capture important audio signal features and translate them to a 2D spectrum image that can help detect cardiac abnormalities. As shown in Figure 3a, normal heart sounds are regular in each heartbeat, while in Figure 3b, an abnormal heartbeat can be observed to occur irregularly.

#### Training and Validation

To train and verify the proposed method, we divided the 3251 recording samples into two datasets: 70% for the training dataset, containing 2276 recordings, and 30% for the validation dataset, containing 975 recordings. The specifications of the computer we used are as follows: 11th Gen Intel(R) Core(TM) i7-11370H @ 3.30 GHz CPU, 16.0 GB RAM, and an NVIDIA GeForce RTX 3070 laptop as the GPU for training the model. Programming was conducted with Python 3.6.8 and TensorFlow 1.15 for the deep learning framework.

**Figure 4:**
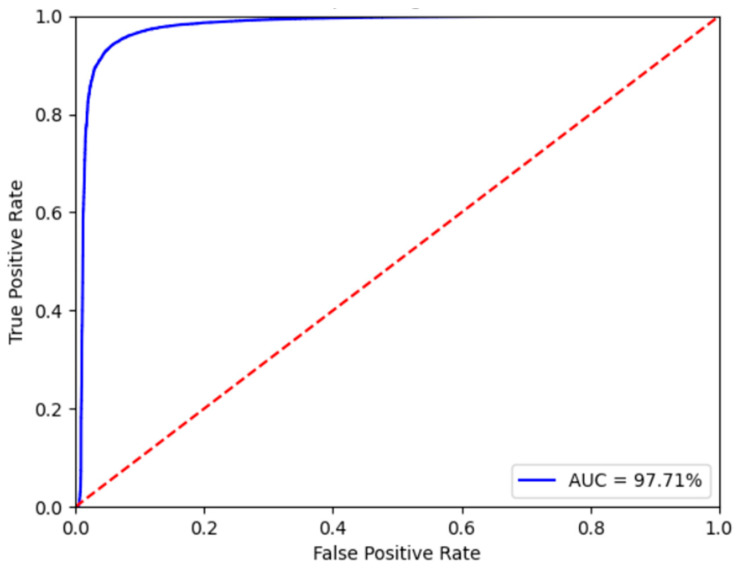
ROC curve of CapsNet on the validation dataset from the 2016 PhysioNet Heart Sound database.

The confusion matrix for the validation result is listed in Table 3. The CapsNet model performs well in predicting the “normal” class with high precision (98.39%) and reasonable recall (84.87%). However, the precision of the “abnormal” class is slightly lower (81.95%), indicating some false-positive predictions. The recall (98.02%) is relatively high, suggesting that the model has a robust ability to identify abnormal samples but may occasionally make errors. Overall, the model’s performance is acceptable, but further improvements may be needed to enhance the accuracy and predictive ability of the “abnormal” class. Figure 4 shows the receiver operating characteristic (ROC) curve, which is a useful tool for evaluating the performance of binary classification models. The curve is generated by plotting different thresholds based on the true positive rate (vertical axis) and the false-positive rate (horizontal axis). This curve is helpful for comparing the performance of models under different thresholds. The CapsNet model has an AUC of approximately 97.71%, which indicates excellent performance. This result demonstrates that the CapsNet model can distinguish between positive and negative samples very well and can maintain a high true positive rate while maintaining a low false-positive rate at almost all thresholds. Therefore, CapsNet achieves exceptional performance in binary classification tasks.

We also compared the results of the training and validation datasets with those of other deep learning network architectures in Table 4. Each model can make good predictions with more than 93% accuracy, but the predictions are lower than 78% on the validation datasets. This may indicate that the CapsNet model does not overfit, which was validated by the strong performance shown in Table 4, with good agreement between the training and validation results. Another benefit is that the training time of CapsNet is lower than that of the other models, except for GoogLeNet. Therefore, CapsNet also has good advantages in terms of training time consumption.

### 3.2. Fine-Tuned Model and Testing with Further Data

#### Hardware Setup and Signal Collection

All the individuals participating in the study were cardiology patients who were aged 0 to 50 and consented to the study. The recorded data were approved by the Institutional Review Board of Taichung Veterans General Hospital (protocol no. CF20047B; date of approval: 11 March 2020). In this case study, physicians labeled cardiac audio signals with a diagnosis by cardiac ultrasound. The collection dataset consists of recordings from 56 patients in clinical auscultation scenarios. The dataset was then split as follows: 75% for the training dataset, containing 42 recordings for fine-tuning purposes, and 25% for the test dataset, containing 14 recordings for final testing.

**Figure 5:**
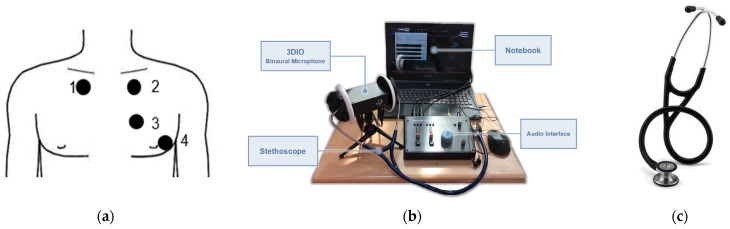
Heart sound recording system: (**a**) Heart sound recording points. (**b**) Stethoscope recording setup. (**c**) Stethoscope.

**Figure 6:**
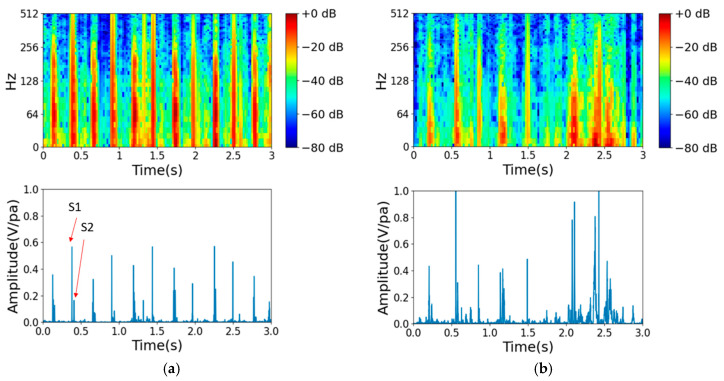
Examples from audio spectrum analysis of heart sounds from our collected test dataset. (**a**) Normal. (**b**) Abnormal.

The heart sounds of each participant were recorded at four different points, as shown in Figure 5a, with each point being recorded for a duration of 15 s. As shown in Figure 5b, the heart sound signal was received by a two-channel 3DIO binaural microphone [32] with a stethoscope. Each signal was recorded in an audio file through an SSL audio sound card. The specifications of the 3DIO binaural microphone are shown in Table 5. The stethoscope has two ends, as shown in Figure 5c; the smaller end is for newborns and children, and the larger end is for adults. For the collected dataset recordings, Figure 6a,b show examples of normal and abnormal audio spectrum analyses of heart sounds, respectively. The first and second heart sounds, represented as S1 and S2, respectively, clearly display the characteristics of heartbeats. In Figure 6a, it is evident that normal heart sounds are regular in each heartbeat, whereas in Figure 6b, a heart murmur can be observed occurring at 2–3 s.

### 3.3. Fine-Tuned Model Results and Discussion

By contemplating the incorporation of a new recording device, the improvement of AI’s diagnostic proficiency in clinical auscultation tests can be realized by refining a pretrained model through the inclusion of Supplementary Data. In this section, we aim to ensure that the model accuracy is robust in clinical auscultation. The recorded signals may have slight variations from the 2016 PhysioNet heart sound signals due to hardware differences. On the other hand, the different heartbeat speeds caused by the short clip lengths may affect the accuracy. Therefore, we fine-tuned the capsule’s parameter settings related to the convolutional layer and segmentation length for potential variations in different heartbeat speeds, as listed in Table 6.

As shown in Table 7, the prediction accuracy is poor when the audio is divided into 1 or 3 s clips. We speculate that the time-frequency characteristics of some slower heartbeat sounds may not allow sufficient time for inclusion in these clips. Alternatively, it is difficult to find the corresponding feature vector due to the low frequency and short length. The model performs best on clips of 5 s. The test accuracy is as high as 91.67%.

Different methods to adjust the learning rate during training were tested, including the ReduceLROnPlateau function, learning rate decay, and fixed learning rate. Table 8 shows that these methods differ in the way that the learning rate is adjusted. The ReduceLROnPlateau function reduces the learning rate based on a specified metric and a criterion for improvement, allowing for adaptive adjustments. Learning rate decay, on the other hand, systematically reduces the learning rate over time, typically by multiplying it by a decay factor at regular intervals. The fixed learning rate, however, keeps the learning rate constant throughout training without any adjustments.

**Figure 7:**
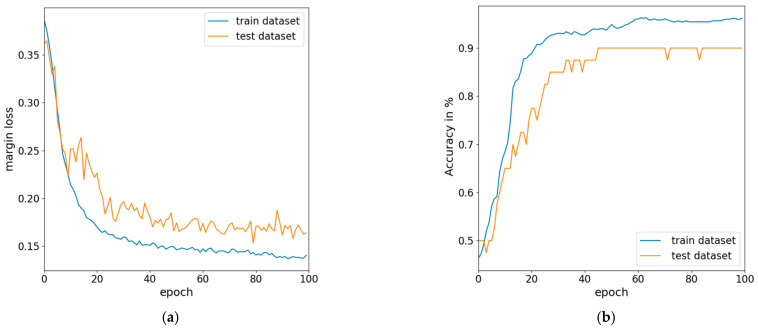
Fine-tuning progress of CapsNet: (**a**) margin loss and (**b**) accuracy.

While a fixed learning rate can work well in certain cases, the other two methods offer adaptability and responsiveness to improve model convergence and achieve higher accuracy. The ReduceLROnPlateau function with learning rate decay leads to the best performance, maintains higher performance by decaying the learning rate, and achieves the highest accuracy on the test set. Additionally, the learning rate decay strategy outperforms the fixed learning rate approach. After fine-tuned testing, we plotted the best settings for the margin loss and accuracy in Figure 7a,b, respectively. We set the early stopping threshold as a 15% difference between the training and testing margin losses to prevent overfitting. Consequently, the result shows successful convergence on the further data.

Table 9 shows a comparison of each model’s training and testing accuracies. The CapsNet model achieves the highest training accuracy of 93.93% and a relatively high test accuracy of 91.67%, demonstrating its good performance in both training and generalizing to unseen test data. The AlexNet model achieves a high training accuracy of 97.56%. However, a significant drop in performance is observed when AlexNet is applied to the test dataset, with a test accuracy of 71.43%. The VGG model achieves a training accuracy of 92.13% and a test accuracy of 78.57%. While the training accuracy is reasonably high, there is some drop in performance on the test set. This may indicate slight overfitting or a need for further optimization. Next, the GoogLeNet model achieves a training accuracy of 91.85% and a test accuracy of 78.57%, similar to the VGG model. It demonstrates comparable training and test dataset performance, suggesting decent generalization. The ResNet model achieves a training accuracy of 75.00%. This result suggests overfitting, where the model memorizes the training data but fails to generalize well to new data.

Compared to conventional CNNs, CapsNet has a significantly different structure and method of processing information. CapsNet has a unique capsule layer structure that enables it to capture spatial hierarchical relationships more effectively and encode possible changes. Thus, CapsNet can preserve the spatial relationships between various parts of an object, while traditional CNNs may lose this information. Moreover, CapsNet’s dynamic routing algorithm allows the model to decide which information to pass to the next layer, making it highly robust against small image changes.

Considering these results when selecting a model for a specific task is important when focusing on balancing high training accuracy and generalization to new data. CapsNet achieves the highest overall performance and high training and test accuracies. Compared to the other deep convolutional neural network models, CapsNet is the only model to achieve convergence.

## 4. Conclusions

This study proposes a CapsNet model for classifying cardiac auscultation and compares various convolutional neural networks to predict normal or abnormal heartbeat sounds. We find that CapsNet can achieve good convergence using only MFCCs as data input, leading to fine-tuned hyperparameters and achieving good performance with a small number of training epochs. The feasibility of CapsNet is demonstrated by its ability to achieve an average test accuracy of 90.29% on the validation dataset from the 2016 PhysioNet heart sound database and 91.67% on the test dataset collected in clinical auscultation scenarios. As a result, the uniqueness and significance of CapsNet lie in its ability to handle the translation equivariance of spectrum features. In contrast, translation invariance in conventional convolution neural networks with pooling falls short of object transformation equivariance. When dealing with the heart sound MFCC spectrum, there is a distinct sound positioning of energy differences between time and frequencies in the spectral images.

Given the limited databases used in this study, the results should be considered preliminary. Therefore, in future works, it is essential to test and calibrate the proposed model on larger databases that include a more extensive range of patients and various rhythm abnormalities to ensure that the proposal is reliable and effective for diagnosing and treating different types of heartbeat abnormalities.

## Figures and Tables

**Table 1:** Detailed parameters for a CapsNet neural network architecture.

Layer (Type)	Output Shape	Trainable Params
input_1 (InputLayer)	(None, 128, 16, 1)	0
conv1 (Conv2D)	(None, 60, 4, 256)	20,992
primarycap_conv2d (Conv2D)	(None, 57, 1, 256)	1,048,832
primarycap_reshape (Reshape)	(None, 912, 16)	0
primarycap_squash (Dynamic routing repeated five times)	(None, 912, 16)	0
digitcaps (CapsuleLayer)	(None, 2, 16)	466,944
Capsnet_Output (OutputLayer)	(None, 2)	0
mask (Mask)	(None, None)	0
capsnet (Length)	(None, 2)	0
decoder (Sequential)	(None, 128, 16, 1)	6,329,344
Total trainable params:		7,866,112

**Table 2:** Default hyperparameter settings for training CapsNet in this study.

Learning_Rate	Epochs	Routings
0.0025	100	5

**Table 3:** Confusion matrix for the validation dataset of the 2016 PhysioNet Heart Sound database.

	Normal	Abnormal	
Normal	2507 (TP)	41 (FP)	98.39% Precision: TP/(TP + FP)
Abnormal	447 (FN)	2029 (TN)	81.95% (TN/(TN + FN))
	84.87% Recall: TP/(TP + FN)	98.02% (TN/(TN + FP))	90.29% Accuracy: (TP + TN)/Total

**Table 4:** Comparison of results for the training and validation datasets.

	Training Accuracy				Validation Accuracy				Training Time (h)
Model	Precision	Recall	F1-Score	Accuracy	Precision	Recall	F1-Score	Accuracy	
CapsNet	97.58%	95.96%	96.77%	96.79%	98.39%	84.87%	91.13%	90.29%	15.7
AlexNet	94.10%	93.74%	93.73%	93.74%	75.14%	73.74%	73.49%	73.91%	18.9
VGG19	98.29%	98.28%	98.28%	98.28%	77.81%	76.88%	76.78%	77.01%	24.3
GoogLeNet	98.56%	97.12%	97.83%	97.82%	75.12%	74.23%	74.01%	74.82%	15.1
ResNet50	98.95%	98.92%	98.95%	98.95%	83.91%	76.31%	74.91%	76.31%	84.3

**Table 5:** Equipment specifications of two-channel microphones.

3DIO Binaural Microphone Equipment Standard	
Frequency response range	60 Hz–20 kHz
Sensitivity	−28 ± 3 dBV/Pa @ 1 kHz RI = 3.9 KHz Vcc = 5 V
Signal-to-noise ratio	80 dB @ 1 kHz
Output impedance	2.4 kΩ ± 30% @ 1 kHz
Operating voltage	2.4 kΩ ± 30% @ 1 kHz
Mic diameter	10 mm

**Table 6:** Capsule parameter settings for CapsNet (layer names are listed in Table 1).

Layer		Filter	Kernel Size	Strides	Batch Size
10 s	Conv1	256	9	2	8
	primarycap_conv2d	16	4	2	
5 s	Conv1	256	9	2	8
	primarycap_conv2d	16	4	1	
3 s	Conv1	128	7	1	16
	primarycap_conv2d	8	4	1	
1 s	Conv1	224	3	1	32
	primarycap_conv2d	32	2	1	

**Table 7:** Comparison of results with different input lengths from the testing datasets.

Segmentation Length	Precision	Recall	F1-Score	Accuracy
10 s	100%	72.72%	84.21%	81.25%
5 s	90.48%	92.68%	91.57%	91.67%
3 s	75.98%	82.88%	79.28%	79.26%
1 s	71.39%	77.56%	74.35%	74.24%

**Table 8:** Effect of different learning rate methods.

	Initial Value	Decay	Unimproved Times	Training Accuracy	Test Accuracy
Reduce learning rate (ReduceLROnPlateau [33])	0.0025	0.15	3	93.93%	91.67%
Learning rate decay	0.005	0.1	-	92.14%	87.50%
Fixed learning rate	0.0025	-	-	92.20%	79.17%

Note: Unimproved times—The learning rate is decreased if the training process does not show a significant improvement in terms of the loss for a certain number of consecutive epochs.

**Table 9:** Comparison of the results on the training and test sets.

	Training Accuracy				Test Accuracy				Training Time (h)
Model	Precision	Recall	F1-Score	Accuracy	Precision	Recall	F1-Score	Accuracy	
CapsNet	94.95%	92.89%	93.91%	93.93%	90.48%	92.68%	91.57%	91.67%	5.2
AlexNet	97.59%	97.52%	97.55%	97.56%	69.77%	73.17%	71.43%	71.43%	6.8
VGG19	93.94%	90.56%	92.23%	92.13%	77.78%	81.40%	79.54%	78.57%	13.4
GoogLeNet	92.93%	90.82%	91.86%	91.85%	80.00%	76.19%	78.05%	78.57%	5.1
ResNet50	90.54%	89.36%	89.28%	89.36%	7812%	75.00%	74.29%	75.00%	15.8

## Data Availability

The 2016 PhysioNet heart sound database used in this study can be found at https://physionet.org/challenge/2016/ (accessed on 10 October 2023).

## Supplementary Materials

Our fine-tuned/test dataset can be found at: https://github.com/fcuyttsai/HeartSoundExtraDataSet/ (accessed on 10 October 2023).
